# 

**Published:** 1820-07

**Authors:** 


### THE LONDON MEDICAL AND PHYSICAL JOURNAL.

CONTAINING

ORIGINAL CORRESPONDENCE OF EMINENT PRACTITIONERS,

AND CRITICAL ANALYSES OF NEW WORKS

RELATING TO

*MEDICINE, SURGERY, CHEMISTRY, PHARMACY, BOTANY, AND NATURAL HISTORY.*

---

*Successively conducted by*

T. BRADLEY, M.D.

A. F. M. WILLICH, M.D.
R. BATTY, M.D.
A. A. NOEHDEN, M.D.
J. ARNEMAN, M.D.

J. P. PFAFF, M.D.

J. ADAMS, M.D.

W. SHEARMAN, M.D.

W. ROYSTON, Esq.

SAMUEL FOTHERGILL, M.D.

AND

WILLIAM HUTCHINSON, M.D.

MEMBER OF THE SOCIETY OF THE COLLEGE OF PHYSICIANS OF PARIS; FELLOW OF THE LINNEAN SOCIETY; MEMBER OF THE MEDICAL AND CHIRURGICAL SOCIETY OF LONDON; AND ONE OF THE PHYSICIANS TO THE ROYAL METROPOLITAN INFIRMARY FOR SICK CHILDREN.

---

VOL. XLIV.

FROM JULY TO DECEMBER, 1820.

Et quoniam variant morbi, variabimus artes;

Mille mali species, mille salutis erunt.

---

*LONDON:*

PRINTED FOR THE PROPRIETORS,

BY J. AND C. ADLARD, 23, BARTHOLOMEW CLOSE;

PUBLISHED BY J. SOUTER, 73, ST. PAUL'S CHURCH-YARD; AND MAY BE HAD OF ALL BOOKSELLERS.

W1
LO 31

### ANALYTICAL TABLE OF THE CONTENTS

OF

THE FORTY-FOURTH VOLUME

OF THE

### London Medical and Physical Journal.

### DESCRIPTIVE ANATOMY.

Page.

Of some points in the structure of the Dugong . . . . 168

Of the human urethra . . . . . . 169, 206

Of the muscles of the bladder . . . . . . 302

Of the lacrymal canals of serpents . . . . . . 347

Of the lymphatics of the intestines . . . . . . 433

Of human entozoa, or intestinal worms . . . . 343, 418

### PHYSIOLOGY.

VITAL PHENOMENA, *in general.*

Reflections on the organic origin of man, and on the nature of vitality . . . . . . . . 43, 46

Views of the general relations of the vital phenomena in different seats . . . . . . . . . 49

NERVOUS AND SENSITIVE SYSTEMS, *in general.*

Observations on the properties of life in the . . . . 51

On the functions of the par vagum . . . . . 82, 125

NUTRITIVE FUNCTIONS (*organic*, of BICHAT).

On the properties and functions of the organs of the, in general . 50

On the means of nutrition in entozoa . . . . 344

*Appetite for, and administration of, food* . . . . . .

*Mastication* . . . . . . . .

*Insalivation* . . . . . . . .

*Deglutition* . . . . . . . .

*Digestion* . . . . . . . .

*Absorption* . . . . . . . .

*Circulation.* On the influence of the arteries and veins on the . 215

On the influence of respiration on the . . . . 333

On the vitality of the blood . . . . . . 47

*Respiration.* On the influence of the par vagum on . . 82, 125

On the mechanism of, especially on the synergy of the glottis and abdominal muscles, and the functions of the diaphragm . . 333

Influence of diseases of the cerebellum on . . . . 73

*Assimilation.* General view of the nature of . . . . 47

On the formation of bone . . . . . . 119

*Secretion* . . . . . . . .

*Excretion.* On the functions of the several parts of the urinary organs 303

CALORIFIC FUNCTION. On the nature and effects of the . . 49

ANIMAL FUNCTIONS.

Remarks on the admitted division of the . . . . . 43

*Sight.* On the relation of the colours of plants to their effects on the system . . . . . . . . 160

*Hearing* . . . . . . . . .

Page

*Smell.* On the effects of some odours on the system . . . 246
*Taste.* On the relations of the taste of substances to their physiological qualities . . . 248
*Touch* . . .
*Sensations* or *Perceptions, in general, and their results.*
On the nature of evidence and the origin of the notion of truth . 37
On the doctrine of the relation of cause and effect . . . 40
*Movements.* On the mechanism of muscular efforts . . . 334
On the power of muscular motion in several nations . . 170
On the action of sphincter muscles . . . 309
Mechanism of the act of vomiting . . . 109, 338
*Sleep* . . .
*Voice* . . .
GENERATIVE FUNCTIONS.
*Preparatory to the act of generation* . . .
*Generative act* . . .
*Gestation* . . .
*Parturition* . . .
*Lactation* . . .
*Sexual characteristics* . . .
PECULIARITIES IN THE FUNCTIONS AT THE DIFFERENT PERIODS OF LIFE.
*Fœtal life.* On the source of . . . 44
*Infancy.* On the peculiar disposition to nervous disorders in . 106
*Childhood* . . .
*Puberty* . . .
*Manhood* . . .
*Old age* . . .
*Death.* Reflections on its nature in general . . . 61

### PATHOLOGY,

GENERAL REMARKS ON.

Abstract view of the nature of disease in general . . . 52

On predisposition to disease . . . 53, 138

OBSERVATIONS RELATIVE TO.

ABSCESS, *urinary* . . . 317

ANEURISM, *carotid* . . . 429

APOPLEXY . . . 370

BLADDER, irritation of the . . . 306

BONES, regeneration of . . . 119

BRAIN, various diseases of the . . . 77

Compression of the . . . 190, 391

BRONCHITIS . . . 221

CATARRH . . . 75

CHOREA . . . 70

CORYZA, *of infants* . . . 427

COW-POX . . . 1, 13, 492

EPILEPSY . . . 116

FEVER, *in general* . . . 16

FEVERS, *epidemic* . . . 132

*eruptive*, in general . . . 16

*gastric* . . . 75

*infantile remittent* . . . 112

*puerperal* . . . 237, 388

*typhous* . . . 76

FISTULÆ . . . 319

GASTRITIS . . . 75, 137

GENITAL ORGANS, diseases of, in general . . . 302

INCONTINENCE OF URINE . . . 304

LEECHES, effects of the bite of . . . 256

NERVOUS SYSTEM . . . 302

[illegible]

| | Page. |
|---|---|
| Perineum, laceration of the | 193 |
| Fungous tumors, of the penis | 74 |
| Hernia | 295, 470 |
| Inflammation. On its nature and causes | 215 |
| On the presence of, in fever | 134 |
| Ophthalmia, in children | 68 |
| in the horse | 118 |
| Prostate gland, inflammation of | 305 |
| Puerperal mania | 155 |
| diseases in general | 63, 231 |
| Rectum, diseases of the | 318 |
| Rubeola | 20 |
| Scarlatina | 18 |
| Small-pox and varioloid diseases | 1, 12, 17, 89, 492 |
| Spinal diseases | 365, 441 |
| Strictures, *of the rectum* | 34, 179 |
| *urethra* | 142, 179, 307 |
| Sympathies, *morbid* | 105, 307 |
| Testicle | 151 |
| Tumors, *of the brain* | 76 |
| Urethra, diseases of, in general | 306 |
| rupture of the | 315 |
| Varioloid diseases | 1, 12, 17, 89, 492 |
| Worms, *intestinal* | 418 |
| Wounds, *thoracic* | 238, 245, 480 |

### PATHOLOGICAL ANATOMY.

| | |
|---|---|
| Of the subjects of fever | 140 |
| puerperal mania | 158 |
| Of a case of fatal stricture of the colon | 180 |
| Of the parts concerned in bronchitis | 227 |
| Of the subjects of mental alienation | 78 |

### NOSOLOGY.

| | |
|---|---|
| Of the Diseases of Women | 236 |
| Children | 450 |

### SEMIOLOGY.

| | |
|---|---|
| Relative to groups of symptoms, or to diseases. | |
| Inflammation and irritation | 64 |
| Some forms of puerperal disease | 65 |
| Bronchitis | 224, 229 |
| Varioloid diseases | 4, 90, 493 |
| Relative to particular Functions. | |
| Those of the mucous membranes | 17, 78 |
| Those of the urinary organs | 307 |

### HYGIENE.

| | |
|---|---|
| Relative to contagious fever | 132 |
| the plague | 296, 338, 490 |

### THERAPEUTICS,

| | Page. |
|---|---|
| GENERAL CONSIDERATIONS ON. | |
| Abstract of the principles of, in general | 58 |
| Remarks on new Remedies | 27 |
| OBSERVATIONS RELATIVE TO. | |
| APOPLEXY | 385 |
| BLADDER, inflammation of the | 306 |
| BRONCHITIS | 224 |
| BRONCHOCELE | |
| CANCER | 265, 353 |
| CHOREA | 70 |
| FARCY, *in the horse* | 83 |
| FEVER | 138 |
| *puerperal* | 237, 388 |
| FISTULÆ, about the anus | 319 |
| GLANDERS, *in the horse* | 82 |
| GONORRHŒA | 153 |
| HERNIA | 295 |
| *humoralis* | 151 |
| INCONTINENCE of URINE | 304 |
| INFLAMMATION, in general | 220 |
| IRRITATION, *of the bladder* | 306 |
| MANIA, *puerperal* | 155 |
| NECROSIS, *of the tibia* | 119 |
| OPHTHALMIA | 72 |
| PERINEUM, laceration of the | 193 |
| POISONS, *vegetable* | 256, 424 |
| PUERPERAL DISEASES | 64, 237, 388 |
| RECTUM, diseases of the | 319 |
| STRICTURE, *of the rectum* | 139 |
| *of the urethra* | 147, 312 |
| URETHRA, rupture of the | 315 |
| WORMS, *intestinal* | 255 |
| WOUNDS, *thoracic* | 238, 289, 490 |
| *gun-shot* | 410 |

### CHIRURGERY,

| | |
|---|---|
| *Respecting the Operations for.* | |
| ARTERIES | 429, 259, 435 |
| BLADDER, puncture of the | 314, 315 |
| FRACTURES OF THE LIMBS, in general | 99 |
| of the lower jaw | 197 |
| HERNIA, *intestinal* | 295, 470 |
| STONES IN THE URETHRA | 348 |
| STRICTURE | 310 |
| TRACHEOTOMY, *in the horse* | 83 |
| URETHRA, diseases of the, in general | 310 |
| WOUNDS, *gun-shot* | 410 |
| *thoracic*, in general | 238, 289, 490 |
| On the use of pressure as a surgical operation | 267, 353 |

### OBSTETRICISM.

| | |
|---|---|
| On some diseases of the puerperal state | 63 |
| Report of the Practice at the Westminster General Dispensary | 231 |
| Instructions for the use of the forceps | 183, 274 |
| Cæsarean operation, case of | 435 |
| On abortion | 234 |

### MEDICAL JURISPRUDENCE AND MEDICAL POLICE.

*Page.*

Statistics respecting the diseases of women connected with procreation . . 231

On the doctrine of the contagion of the plague . . 296, 398, 490

On the influence of medicine on the population of countries . . 401

On the population of the West Indies . . . . . 171

On medical education and practice . . . . . 470

### CHEMISTRY.

On the means of determining the goodness of the dried bulbs of the colchicum autumnale . . . . . . . 285

New tests for copper . . . . . . . 82

Account of some new vegetable alkalies . . . . . 171

On the antiseptic properties of the pyro-lignous acid . . . 172

Crystallization of platina . . . . . . . 351

Oxide of chlorine . . . . . . . . 430

### MATERIA MEDICA, PHARMACY, MEDICAL BOTANY, AND SOME POINTS IN NATURAL HISTORY.

Treatise on the Natural History of Medicines . . . 160, 246

Iodine, recommended for the cure of bronchocele . . . 486

The citrullus cucurbitina, recommended as a remedy for tænia . . 255

The fewillea cordifolia, proposed as a remedy for the effects of vegetable poisons . . . . . . . . 256

Eye-root, proposed for the same purpose . . . . . 434

Observations respecting the colchicum autumnale . . . 285

On the vegetation of bulbs in water . . . . . . 349

Account of the agave americanum . . . . . . 350

Means for preserving the infusion of violets . . . . 431

### MISCELLANEA.

Account of the Royal Metropolitan Infirmary for Sick Children . . 524

Reports of Diseases for London . . 84, 174, 262, 352, 437, 527

On sounds inaudible by certain ears . . . . . 253

On the phenomena experienced on descending in a diving-bell . . 254

Observations on animal magnetism . . . . . 257, 349

Some mineralogical observations . . . . . . . 259

List of autumnal lectures in London . . . . . . 261

Lectures at the School of Physic in Dublin . . . . 348

New theory of electricity and galvanism . . . . . 393

Monthly Catalogues of Books . . 83, 175, 263, 351, 428, 526

Meteorological Journals . . . 87, 176, 264, 439, 440, 526

Bills of Mortality for Paris, for the year 1819 . . . . 80

Prize Questions . . . . . . . 83, 257, 347

Account of the Sirene, a new acoustic instrument . . . . 170

### NAMES OF AUTHORS,

*Of whose Works, Observations, &c. either a detailed Account, or more or less general View, is given in the*

FORTY-FOURTH VOLUME OF THE

### London Medical and Physical Journal.

Page.

ALCOCK, Mr. of *London*, on the Treatment of the Laceration of the Perineum in Parturition . . . . . . . 193
ASPRAY, Mr. of *Olney*, History of a Case illustrative of the Efficacy of Blood-letting in Puerperal Fever . . . . . . 388
BAMPFIELD, Mr. R. W. of *London*, Case and Cure of a very old and irreducible Entero-Epiplocele, with Adhesion to its Sac . . 295
BEDESCHI, Dr. of *Milan*, case of Laceration of the Subclavian Artery . 258
BELL, Mr. CHARLES, of *London*, Treatise on the Diseases of the Urethra, Vesica Urinaria, and Rectum . . . . . . 302
BEUDANT, Mr. some Mineralogical Observations . . . . . 259
BINGHAM, Mr. ROB. of *London*, Practical Essays on Strictures of the Urethra and Diseases of the Testicles . . . . . 142
BOND, Mr. E. T. of *Nun-Eaton*, case of Strangulated Hernia . . 475
BOURDON, Mr. ISIDORE, of *Paris*, on the Mechanism of Respiration, and on the Circulation of the Blood . . . . . . 333
BREMSER, Dr. of *Berlin*, on Living Worms in Living Men . . 427
CHISHOLM, Dr. of *Geneva*, account of some Vegetable Antidotes to Poison 434
CLOQUET, Dr. of *Paris*, on the Lacrymal Canals of Serpents . . 347
COINDET, Dr. of *Geneva*, observations on the Use of Iodine as a Remedy for Bronchocele . . . . . . . . 485
CONFIGLIACCHI, Mr. observations on the Poison of the Viper . . 434
COULOMB, Mr. on the Physical Strength of Men of different Nations . 170
CROSS, Mr. of *Norwich*, History of the Varioloid Epidemic of Norwich . 492
DICKINSON, NODES, Esq. of *London*, Letter to Dr. Grattan, on the State of the Medical Profession . . . . . . . 458
DRAPIEZ, Mr. on the Fewillea Cordifolia, as an Antidote for Vegetable Poisons . . . . . . . . . 256
DUBOIS, Mr. of *Paris*, observations on some Venereal Affections . 74
DUPUY, M. of *Alfort*, observations on the Consequences of Section of the eighth Pair of Nerves in the Horse . . . . . 125
DUPUYTREN, BARON, of *Paris*, account of an Operation on a fractured Jaw 197
ESQUIROL, Dr. of *Paris*, on the Mental Alienation of Puerperal Women and Nurses . . . . . . . . 155
EVANSON, Dr. of *Cork*, case of unusual Determination of Blood to the Head 379
FOHMANN, Mr. of *Heidelberg*, account of a new Discovery respecting the Lymphatic Vessels . . . . . . . 433
GENSANA, Dr. of *Saluzzo*, account of a Case in which the Cæsarean Operation was performed with a favourable event . . . 435
GRANVILLE, Dr. A. B. of *London*, Report of the Practice of Midwifery at the Westminster General Dispensary, in the year 1818 . . 231
Remarks on the Use of the Forceps in Midwifery . . . 274
Proposal of a Nosological Classification of the Diseases of Children 450
GUERBOIS, Mr. of *Paris*, observations on some Venereal Affections . 74
HALL, Dr. MARSHALL, of *Nottingham*, on a severe Puerperal Affection . 63
HAMEL, Dr. of *Petersburg*, on the Effects of a Descent in the Sea in a Diving-Bell . . . . . . . . 254

Page.
HARTY, Dr. of *Dublin*, Historic Sketch of the Causes, Progress, Extent, and Mortality, of the Contagious Fever epidemic in Ireland in 1817, 1818, and 1819 . . . . . . 401
HARRISON, Dr. EDWARD, of *London*, fatal case of Variola post Variolam Vaccinam . . . . . . . . . 12
Remarks upon the different Appearances of the Back, Breast, and Ribs, in Persons affected with Spinal Diseases; and on the Effects of Spinal Distortion on the Sanguineous Circulation . . 365
Cases of Spinal Disease . . . . . . 441
HARRISON, Dr. RICHARD, of *London*, Essay towards a Pathology of Eruptive Fevers . . . . . . . . . 16
Observations and Reflections on the Sympathies existing between the Stomach and Intestines with the Brain . . . 105
HASTINGS, Dr. of *Worcester*, Treatise on Inflammation of the Mucous Membrane of the Lungs . . . . . . 214
HAYES, Mr. E. his Examination before a Committee of the House of Commons respecting the Contagion of the Plague . . . 393
HEBREART, Dr. of *Paris*, on some Diseases of the Cerebrum, Cerebellum, &c. 77
HICKS, Mr. of *Bath*, on the Treatment of Stricture of the Rectum . 34
HOME, Sir E. Bart. of *London*, Microscopical Observations of the Human Urethra . . . . . . . . 169
HUTCHISON, Mr. A. COPLAND, of *London*, observations on the Subject of the proper Period for Amputating in Gun-shot Wounds . . 410
Remarks on an extraordinary Disease in the Eye of the Horse . 118
JOHNSON, Dr. JAMES, of *London*, Treatise on the Derangements of the Liver, Internal Organs, and Nervous System . . . 322
JADELOT, Dr. of *Paris*, on an Epidemic Inflammation of the Globe of the Eye in Children . . . . . . . 70
KINGLAKE, Dr. of *Taunton*, on the Efficacy and Fallacy of new Remedies 27
On the Position of Fractured Limbs . . . . 99
LALLEMAND, Mr. of *Paris*, Chirurgical Observations . . . 76
LARREY, BARON, of *Paris*, cases of Wounds penetrating the Thoracic Cavity, with some Remarks on their Consequences, and on the Effects of Paracentesis of the Thorax for Empyema . 246, 288, 480
LEBEL, Dr. of *Paris*, Reflections on the Generation of Bones . . 119
LESAGE, Dr. of *Paris*, account of a severe Wound in the Perineum . 390
MAC GRIGOR, Sir JAMES, his Examination before a Committee of the House of Commons, respecting the contagious Nature of the Plague 297
MACLEOD, Dr. of *London*, Remarks on the Small-pox, as it occurred in London subsequent to Vaccination . . . . 1
On the common Origin of all Varioloid Diseases . . . 89
MONGENOT, Dr. of *Paris*, principal Facts observed at the Hospital for Sick Children during the year 1815 . . . . 68
MURRAY, Mr. various Botanical Observations . . . . 340
PINEL, Prof. of *Paris*, General Remarks on the Medical Constitution of the Decline of the Summer and the Autumn of the year 1815 . 75
PRICHARD, Dr. of *Bristol*, History of the Epidemic Fever which prevailed in Bristol during the years 1817, 1818, and 1819 . . 132
PRING, DANIEL, Esq. of *Bath*, General Indications which relate to the Laws of the Organic Life . . . . . . 36, 128
RAFFLES, Sir STAMFORD, of *Ceylon*, account of the Dugong . . 168
RAMSAY, Mr. W. on the Antiseptic Powers of the Pyro-lignous Acid . 172
RAYER, Dr. of *Paris*, observations on the Coryza of young Infants . 427
RUDOLPHI, Prof. of *Berlin*, Synopsis of the Entozoa . . . 342
SHAW, Mr. of *London*, on the Structure of the Membranous Part of the Urethra . . . . . . . . . 206
TAINSH, Dr. his Examination before a Committee of the House of Commons, respecting the Contagion of the Plague . . . 490
THOMSON, Mr. TODD, of *Sloane-street*, account of some Experiments to prove the Advantage of employing the Alcoholic Solution of Guiaic as a Test of the Goodness of the dried Bulbs of the Colchicum Autumnale . . . . . . . . . 282

*Page.*
THOMSON, Dr. of *Edinburgh*, observations on Varioloid Diseases . . 492
VACCA, Prof. of *Pisa*, on the Ligature of Arteries . . . . . 429
VIREY, Dr. of *Paris*, Natural History of Medicines, Aliments, and Poisons 160, 246
WALKER, Mr. R. of *Oxford*, observations on Venereal Diseases . . 472
WALTHER, Prof. of *Bonn*, case of Ligature of the Carotid Artery . 435
WHITE, Mr. W. of *Bath*, case of Stricture of the Colon, terminating fatally 179
WOLLASTON, Dr. of *London*, observations on Sounds inaudible by certain Ears 253
YOUNG, Mr. SAMUEL, of *London*, a Series of Cases exhibiting the Effects of Pressure in Cancerous and other Diseases . . 265, 353

### MONTHLY CATALOGUE OF MEDICAL BOOKS.

A Treatise on Inflammation of the Mucous Membrane of the Lungs: to which is prefixed, an Experimental Inquiry respecting the Contractile Power of the Blood-vessels, and the Nature of Inflammation. By Charles Hastings, M.D. 8vo. 10s. 6d.

Advice and Maxims for Young Students and Practitioners of Medicine; with Remarks on the Pulse. By Daniel Johnson. 1s. 6d.

A Treatise on the Mineral Water of Askern, Yorkshire. By T. Le Gay Brewerton, Surgeon. 8vo. 5s. 6d. boards.

Cases of a Serious Morbid Affection, principally incident to Females after Delivery, Abortion, &c. and arising from Uterine Hemorrhagy, undue Venesection, Menorrhagia, protracted Lactation, Diarrhœa, Aphthæ, Constipation, Scybalæ, or other Causes of Exhaustion and Irritation. By Marshall Hall, M.D. F.R.S.E. &c. 8vo. 4s.

Observations on Strictures of the Rectum, and other Affections which diminish the Capacity of that Intestine. By W. White, Esq. Member of the Royal College of Surgeons, London, &c. Third Edition. 7s. 6d.

Principles of Military Surgery; comprising Observations on the Arrangement, Police, and Practice, of Hospitals; and on the History, Treatment, and Anomalies, of Variola and Syphilis, illustrated by Cases, Dissections, and Engravings. By John Hennen, M.D. F.R.S.E. Second Edition, with numerous Additions. 8vo. 18s. boards.

### TO CORRESPONDENTS.

Adjutor *will find, that the subject of his Letter has been treated of in the present Number, and that what he has only stated generally has been discussed in a particular manner. If he will send us a similar account of his observations, we shall certainly appropriate them for insertion in our Journal: such a way of considering the subject, is the only one that can produce useful results. Still, we should be inclined to publish his judicious suggestions, were it not that popular prejudice is already turning with some violence against the measure alluded to in them, because it has not effected* all *that it was expected to do; and we are, at present, too warm friends to the use of the measure in question, to favour in any way this prejudice, by giving publication to remarks that will add to the alarm without elucidating the object of enquiry.* Adjutor, *we have no doubt, will acknowledge the propriety of our conduct in this instance. We hope, therefore, that our decision respecting his last Paper will not prevent our receiving future Communications from him. We should add, that, should he favour us with a particular account of the subject of that paper, we request he will make us acquainted with his name, though we shall not insist on the publication of it; as, on such a subject we must know whose authority we have for what we sanction by inserting in our Journal.*

*We shall give a place to* R. S.*'s paper as soon as the order of its reception will permit; chiefly because the object of it is to promote enquiry on an interesting subject, and because we would cherish such conduct in a young student (such we judge him to be), as is evinced in his paper. But we would inform him, that we think he has taken too exclusive a view of the opinions of the parties which he alludes to. Teachers, in their Lectures, often seize on certain points, on which they have some new hypothesis or theory, and seem to dwell exclusively on them; but their object is only to elucidate what they consider to be obscure, and yet of most importance; whilst they neglect to notice minor circumstances, only because they suppose that every one is already well acquainted with them, and will necessarily take them into consideration. This hint may be useful to* R. S. *in the course of his studies. He need not have refrained from putting the signature of his name to his paper: the Lecturer whose opinions he especially critises would, we are confident, be pleased to observe such conduct; for, next to his desire to convey necessary information to his pupils, is that of leaving them to think for themselves in the formation of their opinions; and he usually throws out many hints in his discourses especially directed to such an object.*

*The writers of anonymous Communications should let us know how we can address them privately. We have not sufficient space to permit us to give our reasons for rejecting several; and a simple statement that they are rejected, will, in many instances, convey erroneous ideas of our opinions respecting their merit.*

